# Predicting Postoperative Vision for Macular Hole with Automated Image Analysis

**DOI:** 10.1016/j.oret.2020.06.005

**Published:** 2020-12

**Authors:** Declan C. Murphy, Amar V. Nasrulloh, Clare Lendrem, Sara Graziado, Mark Alberti, Morten la Cour, Boguslaw Obara, David H.W. Steel

**Affiliations:** 1 Newcastle University, Newcastle Upon Tyne, United Kingdom; 2 Northumbria Healthcare NHS Foundation Trust, Tyne and Wear, United Kingdom; 3 Department of Computer Science, Durham University, Durham, United Kingdom; 4 Physics Study Program, University of Lambung Mangkurat, Banjarbaru, Indonesia; 5 National Institute for Health Research Newcastle In Vitro Diagnostics Co-operative, Newcastle University, Newcastle Upon Tyne, United Kingdom; 6 National Institute for Health Research Newcastle In Vitro Diagnostics Co-operative, Newcastle upon Tyne Hospitals Foundation Trust, Newcastle Upon Tyne, United Kingdom; 7 Department of Ophthalmology, Rigshospitalet, University of Copenhagen, Copenhagen, Denmark; 8 Sunderland Eye Infirmary, Sunderland, United Kingdom

**Keywords:** AL, axial length, BD, base diameter, MH, macular hole, MLD, minimum linear diameter, SD-OCT, spectral domain OCT, 3D, 3-dimensional, VA, visual acuity

Idiopathic macular holes (MHs) are routinely treated with high surgical closure rates. Although successful hole closure is the chief determinant for postoperative best-corrected visual acuity (VA), the extent of visual improvement achieved in successfully closed MHs after surgery is variable.^1^

Size of MHs is typically defined by its minimum linear diameter (MLD) on spectral domain OCT (SD-OCT), which is predictive of postoperative outcome.^2^ However, current methods that measure the MLD of MHs are prone to high interobserver and intraobserver variability and inaccuracies. Minimum linear diameter also does not account for the recognized asymmetry in MH shape and overall size.^3^ Other measures that assess MH size and shape have been suggested as better predictors of postoperative vision, including those based on 3-dimensional (3D) measures.^4^^,^^5^

Previous studies have been restricted in their ability to precisely determine the extent by which MH size can predict postoperative vision outcomes due to numerous limitations, including inaccurate VA and size measurements, variable surgical interventions, and cataract.

We developed a fully automated 3D image analysis methodology that accurately measures MHs using SD-OCT scans.^6^ In this study, we used data from a previously reported prospective randomized controlled trial to predict postoperative VA after successful MH surgery.^7^ We hypothesized that automated measures of MH size would provide more accurate predictions of postoperative vision. Data were obtained from a previously published randomized controlled trial that obtained ethical approval (protocol Number: H-4-2013-091, Rigshospitalet, Copenhagen) and full informed consent from all participants. All research adhered to the tenets of the Declaration of Helsinki.

All participants were pseudophakic before surgery and underwent the same surgical procedure. Visual acuity was determined using the Early Treatment Diabetic Retinopathy Study protocol with refraction at baseline and 3 months after surgery. Participants were randomized to face-down or nonsupine positioning for 3 days. There was no difference in closure rate or VA between groups, and closure was achieved after the first surgical intervention in 67 of 68 participants.

**Figure 1:**
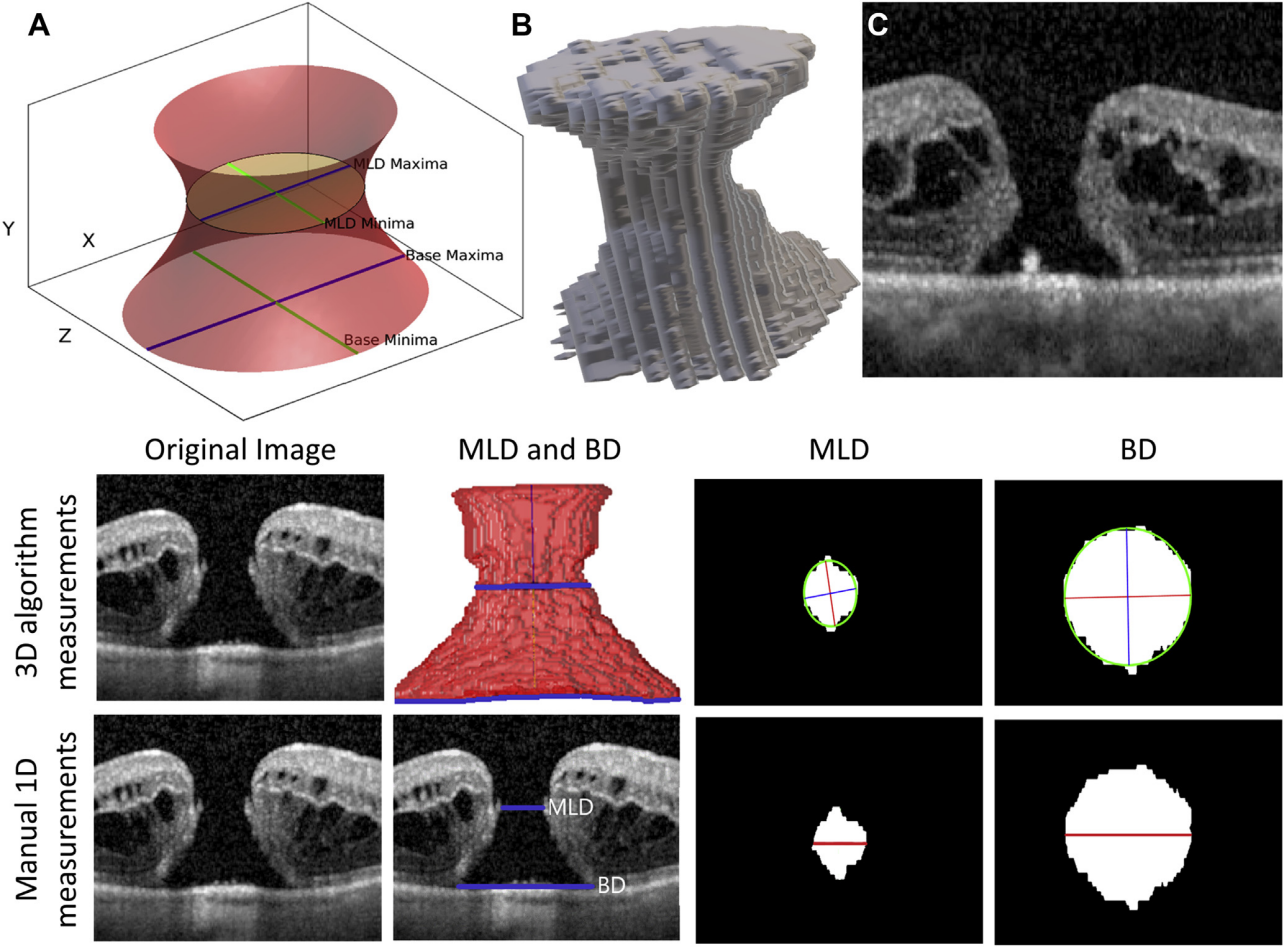
Measurements of macular hole (MH) using a 3-dimensional (3D) algorithm. **Top:** Representation of how the 3D model was used to measure 3D parameters. **A,** Diagrammatic representation of an MH including annotations that represent several MH measurements: Algorithm-derived measurements included height (computed by a smooth centerline from the center of the MH base to its top area), the maximum and minimum dimensions of the base area (BDmaj and BDmin), base area (BA), the maximum and minimum dimensions of the minimum area (defined as the minimum area in the central 20%–90% of the hole height) (MLDmaj and MLDmin), minimum area, the maximum and minimum dimensions of the top area (TDmaj and TDmin), top area, surface area, and volume. Mean diameters were taken as the mean of the maximum and minimum measurements. Four previously described size ratios were calculated: macular hole index (height/mean BD), tractional hole index (height/MLDmin), diameter hole index (mean MLD/mean BD), and area ratio factor (surface area – [top area + BA])/BA). **B,** Three-dimensional representation of MH reconstructed using the automated 3D algorithm. **C,** Spectral-domain OCT (SD-OCT) single-slice image of MH which corresponds to the 3D model in **(B)**. **Bottom:** Example of differences between clinician-derived 1-dimensional MH measurements and 3D algorithm-derived measurements. **Right:** The outputted values for the algorithm-derived minimal and base areas (minimum axes in blue and maximum in red), and the clinician manual MLD and BD (both in red) are shown. In this case, the algorithm MLDmin was 355, MLDmaj 390, human MLD 395, BDmin 782, BDmaj 1033, and human BD 955 μm.

Recorded variables include age, gender, axial length (AL), symptom duration, randomization group, and clinician-measured base diameter (BD) and MLD. All patients underwent SD-OCT (Heidelberg Spectralis, Heidelberg, Germany) before and 3 months postoperatively using a 15-by-5-degree block, with 49 horizontal scans at 30-μm spacing. The automated multiscale 3D level set segmentation approach was as previously described, with an accuracy of segmentation of 99.19% compared with a ground-truth manual segmentation approach by an experienced clinician.^6^ All images were checked for gross segmentation errors, and cases with a discrepancy of >10% from clinician BD measures were evaluated for segmentation errors. A range of size parameters were produced using the 3D image analysis algorithm, herein referred to as “algorithm-derived measurements”^3^ (Fig 1).

To assess for scaling errors secondary to variable magnification, we corrected the lateral scale of the OCT datasets for inter-individual differences in AL and compared them with measurements without. Generalized linear modeling using a forward/backward stepwise procedure was used to model postoperative VA.

A total of 67 eyes of 67 participants with primarily closed MHs were analyzed. Patient parameters are described in Table S1 (available at www.ophthalmologyretina.org). Visual acuity improved from a mean of 51 preoperatively to 71 Early Treatment Diabetic Retinopathy Study letters postoperatively. Mean MLD was 380 μm, and 46% were greater than 400 μm. Median symptom duration before surgery was 7 months, and 16% had symptoms for 12 to 24 months. No images showed segmentation errors.

The correlations between preoperative and postoperative VA and algorithm-derived measurements are shown in Table S2 (available at www.ophthalmologyretina.org). Surface area was most highly correlated with preoperative vision, with a correlation coefficient (r) of 0.74. Preoperative vision was highly correlated with postoperative VA (r=0.64). The most predictive model of postoperative VA was based on preoperative VA and MH height, with the following regression formula:

Postoperative VA =32+ (0.6×Preoperative VA) + (0.02×height)

R^2^ was 45% with approximately 40% of variability explained by preoperative VA (*P* < 0.0001) and 5% by height (*P* = 0.027). It is important to note that MH height was computed by a smooth centerline from the MH base center to its roof, which differs from a perpendicular height commonly measured in 1 dimension in current clinical practice.^3^

This model used an accurate measurement of preoperative VA. In routine clinical practice, it can be difficult to obtain this for practical reasons. When preoperative VA was excluded, the best prediction of postoperative VA was as follows:

Postoperative VA = 37 - (0.05 × MLDmaj) + (0.05 × TDmaj) + (1.3 × AL)

R^2^ was 35% with 19% explained by *MLDmaj* (*P* < 0.0001); 10% by *TDmaj* (*P* = 0.0025); 6% by AL (*P* = 0.045).

Although the clinician MLD and BD had similar levels of correlations with the preoperative and postoperative visual acuities as the algorithm-derived ones, these measures are less accurate; the most predictive model without the algorithm values had a predictive value of just 20% and included MLD only.

Postoperative VA = 80 - (0.02×clinician MLD);R2 =20%,P= 0.0002.

Adjusting scan measurements to absolute measures of MH size using AL did not improve the predictive ability of our models (Table S3, available at www.ophthalmologyretina.org). It should be noted that the MLD is also still of high value in predicting closure, which we did not assess.^1^

We did not find an association between MH duration before surgery and visual outcomes, which may be due to the relatively long duration in this study. Likewise, age was not associated with postoperative vision, perhaps because age-related macular degeneration and other pathology were excluded in our cohort.

Previous groups have studied the 3D MH parameters using different methodologies.^4^^,^^5^ Xu et al^5^ also used an automated technique, but in distinction to their system, the one we have developed considers the overall 3D geometry of the hole, uses a unique surface cutting algorithm, is significantly faster, and is more robust to image artefact. They found associations between visual outcome and various size parameters, including volume; however, their study was limited by several potential clinical confounders.^5^

This study has limitations. We only assessed closed holes, which limits generalizability. Follow-up was limited to 3 months; thus, results may not be valid at longer time periods after surgery. There are other morphologic variables that we did not include, such as ELM height and outer retinal integrity, which could be combined with our methodology in future studies.
