# Supplementary material for: Predicting Postoperative Vision for Macular Hole with Automated Image Analysis
Source: Ophthalmol Retina. 2020 Dec;4(12):1211–3. doi: 10.1016/j.oret.2020.06.005 (PMC7720681; doi:10.1016/j.oret.2020.06.005)
Supplement: Table S1 [file mmc1.pdf]

	Variable	Result
**Patient characteristics**		
**All variables reported as mean; range; SD**	Age in years	69.8; 48.0-81.0; 6.1
	Pre-operative *VA* (ETDRS letters)	51.4; 35.0-73.0; 8.7
	Post-operative *VA* (ETDRS letters)	71.3; 54.0-86.0; 6.9
	Axial length (mm)	23.8; 21.6-26.8; 1.1
**All variables reported as N (%)**	Sex	Male: 27 (40.3)<br>Female: 40 (59.7)
	Presence of VMT	Yes: 10 (14.9)<br>No: 57 (85.1)
**Pre-operative algorithm-derived measurements**		
**All variables reported as mean; range; SD**	*SA* (mm^2)	2.2; 0.4-4.7;0.9
	DHI	0.4; 0.1-0.9; 0.1
	MLDmin (microns)	379.6; 47.8-704.0; 140.1
	MLDmaj (microns)	433.9; 94.5-641.5; 150.9
	TDmaj (microns)	554.4; 275.6-806.8; 119.2
	TDmin (microns)	481.1; 245.7-699.1; 105.0
	TA (mm^2)	0.2; 0.1-0.4; 0.1
	Height (microns)	425.4; 206.5-538.1; 69.0
	BDmaj (microns)	987.8; 198.3-1561.6; 274.6
**All variables reported as**	*Volume* ($10^{-3}mm^3$)	106.4; 8.0-327.5; 67.9-143.3
	MHI	0.5; 0.3-1.4; 0.4-0.5
	THI	1.1; 0.5-6.9; 0.9-1.4

**median; range; IQR**	*BA* (mm^2)	0.7; 0.0-2.1; 0.5-0.9
	MA (mm^2)	0.1; 0.0-0.4; 0.1-0.2
	ARF	1.8; 1.0-9.3; 1.8-2.1

Supplementary table 1: Patient characteristics and algorithm-derived measurements. Describes baseline characteristics of the study cohort and pre-operative macular hole measurements derived using the automated three-dimensional algorithm. Results are based on 67 participants.

Abbreviations: *ARF*: area ratio factor; *BA*: base area; *BDmaj*: *Base diameter* (largest measurement); *DHI*: Diameter hole index; ETDRS: Early Treatment Diabetic Retinopathy Study; IQR: interquartile range; *MA*: minimum area; *MHI*: macular hole index; *MLDmaj*: minimum linear diameter (largest measurement); mm: millimeters; *MLDmin*: minimum linear diameter (smallest measurement); mm: millimetre; *SA*: surface area; SD: standard deviation; *TA*: top area; *TDmaj*: top diameter (largest measurement); *TDmin*: top diameter (smallest measurement); *THI*: tractional hole index; *VA*: Visual acuity; VMT: Vitreomacular traction;
