# Supplementary material for: Predicting Postoperative Vision for Macular Hole with Automated Image Analysis
Source: Ophthalmol Retina. 2020 Dec;4(12):1211–3. doi: 10.1016/j.oret.2020.06.005 (PMC7720681; doi:10.1016/j.oret.2020.06.005)
Supplement: Table S2 [file mmc2.pdf]

Variable	Correlation with pre-operative vision (R)	Correlation with post-operative vision (R)
**DERIVED FROM 3D ALGORITHM MEASUREMENTS**		
Height	-0.39	-0.06
MLDmaj	-0.71	-0.45
MLDmin	-0.68	-0.43
BDmaj	-0.73	-0.40
BDmin	-0.73	-0.42
Volume	-0.72	-0.36
SA	-0.74	-0.38
MHI	0.60	0.45
THI	0.37	0.25
DHI	-0.14	-0.11
TDmaj	-0.61	-0.30
TDmin	-0.62	-0.33
BA	-0.69	-0.37
TA	-0.32	-0.32
**DERIVED FROM CLINICIAN MEASUREMENTS**		
MLD	-0.70	-0.44
BD	-0.73	-0.42

Supplementary table 2: Macular hole measurements and their correlation with pre-operative and post-operative vision. Describes pre-operative MH measurements and their association with pre-operative vision. All results are calculated using 67 participants. Abbreviations: *BA*: Base area; *BD*: Base diameter

(clinician-derived measurement in the horizontal plane); *BDmaj*: base (largest measurement); *BDmin*: base diameter (smallest measurement); *DHI*: diameter hole index; *MHI*: macular hole index; *MLD*: minimum linear diameter (clinician-derived measurement in the horizontal plane); *MLDmaj*: minimum linear diameter (largest measurement); *MLDmin*: minimum linear diameter (smallest measurement); *SA*: surface area; *TA*: Top area; *TDmaj*: Top diameter (largest measurement); *TDmin*: Top diameter (smallest measurement); *THI*: tractional hole index.
