# Supplementary material for: Predicting Postoperative Vision for Macular Hole with Automated Image Analysis
Source: Ophthalmol Retina. 2020 Dec;4(12):1211–3. doi: 10.1016/j.oret.2020.06.005 (PMC7720681; doi:10.1016/j.oret.2020.06.005)
Supplement: Table S3 [file mmc3.pdf]

Parameter	Unadjusted Size		Adjusted Size	
	**Post-operative VA (R)**	**Pre-operative VA (R)**	**Post-operative VA (R)**	**Pre-operative VA (R)**
Height	-0.06	-0.39	-0.06	-0.39
MLDmaj	-0.45	-0.71	-0.42	-0.70
MLDmin	-0.43	-0.68	-0.42	-0.67
BDmaj	-0.40	-0.73	-0.38	-0.72
BDmin	-0.42	-0.73	-0.41	-0.72
Volume	-0.36	-0.72	-0.34	-0.72
SA	-0.38	-0.74	-0.35	-0.73
TDmaj	-0.30	-0.61	-0.24	-0.59
TDmin	-0.33	-0.62	-0.33	-0.61
BA	-0.37	-0.69	-0.36	-0.69
TA	-0.32	-0.62	-0.29	-0.61

Supplementary table 3: Correlations of measured parameters with pre- and post-operative vision using the three-dimensional automated algorithm method with and without adjusted measurements based on axial length.

Abbreviations: *BA*: Base area; *BDmaj*: base diameter (largest measurement); *BDmin*: base diameter (smallest measurement); *MLDmaj*: minimum linear diameter (largest measurement); *MLDmin*: minimum linear diameter (smallest measurement); R: correlation coefficient; *TA*: Top area; *TDmaj*: Top diameter (largest measurement); *TDmin*: Top diameter (smallest measurement); VA: visual acuity.
